# Australian sport policy and advocacy: an historical account of policy evolution, government involvement, and the advent of lobbying

**DOI:** 10.3389/fspor.2025.1656342

**Published:** 2026-01-15

**Authors:** Charles Mountifield

**Affiliations:** University of Canberra, Canberra, ACT, Australia

**Keywords:** government priorities, grassroots, health, policy influence, fitness

## Abstract

Various factors have influenced the evolution of Australian sport policy, including war and the importance of national health. Historically, the involvement of the Australian federal government was sporadic, and policy decisions were subject to prevailing political agendas. Within that context, moves toward consistent sport policy were influenced by the initially indirect and, more latterly, direct influence of lobbying. This paper explores the processes that shaped Australian sport policy, including the burgeoning role of advocacy. Examining sport as government policy, the paper is organised into four thematic sections, commencing with background on policy, followed by an outline of sport policy post-federation leading up to the early 1970s, then a review of developments post-1970, and concluding with an outline of the development of lobbying. Methodologically, literature, including journal articles, books, government documents, Hansard, and “grey” material, formed the basis of a narrative review to illuminate the four themes. The findings point to an evolutionary process that elevated sport to a significant policy consideration, with various elements such as fitness, health, and international sporting success becoming essential to the architects of sport policy. Within that framework, the role of advocacy developed to significant effect but more recently, lobbying in sport policy has become subject to the vicissitudes of political interest and commercial influences, creating a void in the policy process.

## Introduction

The evolution of Australian sport policy since the federation has been gradual, inadvertent, and subject to differing government interests and investment levels. Indeed, in the first forty years of federation at both federal and state levels, governments had not established policies that considered how sport might play a role in national or community development (1). Equally, there was an absence of a clear understanding of the significance of sport and fitness and the associated benefits to the nation's health (2, 3). In the years from federation leading up to World War II, sport policy in Australia was not considered an area for overt government involvement, but the advent of war provided the impetus for a policy change (4–7). In the post-war years through to the 1970s, government connection with sport policy was erratic and distant (3, 8–14). From the 1970s, government involvement in sport policy became more direct and calculated (3), and was followed by the advent of lobbying for sport in the 1980s (15).

Noting the above context, this paper outlines the historical development of sport policy in Australia, the vicissitudes of government interest and investment, the gradual institutionalisation and commercialisation of sport, and describes the role of advocacy in sport. Critical influences on sport are discussed, including government and National Sport Organisation (NSO) foci on national sporting prowess on the international stage (13, 16, 17), the increasing levels of commodification and commercialisation of sport (18, 19), the consideration of the benefits of sport on the broader national health (5, 7, 20), factors affecting various stakeholders in the Australian sport ecosystem (3), resistance to reviews of sport policy (21), and the influence of advocacy groups over time (20, 22). While the advent of lobbying played a significant role in developing sport policy, however, the influence of advocacy groups has waned.

### Significance

From the perspective of history and the advent of lobbying, advocacy groups invariably evolve where there is a need to influence policy and address a power imbalance between stakeholders (23–25). There are numerous factors that impact policy processes that can be found in political arrangements, including federalism, bureaucratic structures, and systems for interest intermediation systems, where social groups outline collective interests to government (26). Understanding policy processes suggests that the combination of institutional and conceptual transformation can affect the policy-making process and result in policy change (27). Although collective approaches to policy are positive for communities, many community organisations fail to operate effectively in the public policy space, leading to tensions based on local service implementation (28). The indication is that collaboration between government, community service organisations, and local communities is chaotic and needs modification in order to fulfil social objectives (28). Such observations are likely to be similar for grassroots sport which, where the concept of health and participation for all is concerned, forms an integral part of the social focus (29, 30).

Despite the potential for history to provide a platform for learning (31), however, there are distinct variations in policy processes relating to participatory sport (32). Further, an historical account of Australian sport policy unearths the unpredictable and inconsistent nature of government investment in physical activity through sport (7, 32), from both a conceptual and pecuniary basis. Indeed, a degree of rhetoric concerning responsibility for the various levels of sport persists to this day (33–36). Sport policy research has attracted increasing attention (37), led to calls for better frameworks for sport policy (38, 39), and consideration of the ever-increasing commercial pressure on sport (21, 40, 41). Within that context, however, lobbying for sport has become decentralised, much maligned, and subject to NSO and government political agendas.

Thus, this paper seeks to outline the historical development of lobbying in Australian sport, whilst noting that the impact of advocacy groups is minimal and sport policy is very much a top-down process (42, 43). The significance of the situation is such that achieving a balanced approach to sport policy is challenging, especially when trying to link advocacy and general health through sport (7, 44). To understand the influences on the policy process for the broader benefit of all Australians, there is a need for comprehension of the evolution of sport policy in Australia and the importance of advocacy. This research provides insight into that objective and seeks to answer the following question: *What do academic sources and grey literature identify as factors influencing the evolution of Australian sport policy since federation and the more recent development of advocacy in sport?*

## Method

The paper follows a qualitative approach for the analysis of the literature herein, noting that the simplest qualitative literature review is “often referred to as the *narrative review”* (45). The narrative review format is common in sport-related research [see (46–49)], including that concerning sport policy [see (43)]. Narrative reviews outline connected events and describe research topics from a largely contextual position but generally do not allow for the reproduction of data nor address quantitative objectives (50). Such reviews consist of a process for summarising aspects of a topic (45) and adopting a structure that introduces a topic, outlines the development in a narrative format, and concludes with a discussion element (50). In addition, the paper provides a section that provides recommendations for further research. Ultimately, with regard to the policy focus of this research, the objective is to provide an outline of literature to help deepen understanding based upon a purposive selection of evidence (51).

### Data collection

A range of literature was reviewed to provide the data for developing the topic due to the variety of sources providing information on the subject. The review considered journal articles, books, government documents, Hansard, and other grey literature. Owing to the nature of the review documents, the data was collected based on simple searches through Google Scholar, academic institution libraries, government websites, and websites in general. Owing to the wide variety of sources, no formal approach (e.g., systematic review or the use of Boolean operators) was applied to gathering data. Instead, the data was selected based on the author's knowledge of the field and on a non-exhaustive basis, key search terms included (1) history of Australian sport; (2) Australian sport policy development; and (3) advocacy in Australian sport. No timeframe was set, largely due to the extent of the period covered by this review, and to try to capture as much information as possible.

### Data analysis

In keeping with the relative subjectivity of narrative reviews (52), the data was evaluated by the author and manually grouped into the following themes: (1) Policy Background, (2) Sport Policy Pre-1970, (3) Sport Policy Post-1970, and (4) Sport Policy and Advocacy. This thematic allocation was based on the author's initial objective for the paper, followed the interpretation of findings relevant to the study, and the overall objective of outlining a “narrative” that informed the research objective.

### Delimitation

The intent and purpose of this paper was to provide an outline of sport policy development in Australia and the advent of advocacy, based on the flexible nature of a narrative literature review. Narrative reviews are not designed to be conclusive but instead provide a platform for further research (45). Unlike the stricter guidelines governing systematic reviews, for example, narrative reviews have fewer rules (53). Researchers decide on the review's focus and what will be included to offer valuable research (54). Indeed, such literature reviews are almost always selective, non-exhaustive, and involve less rigorous search methods and ways of highlighting grey literature (45). Further, narrative reviews do not require systematic recording of data which is acknowledged as a limitation but a matter that can be accommodated with further research on the topic [e.g. (49),].

## Literature review

Reflecting on the reference to themes, the narrative review is divided into four thematic sections consisting of Policy Background, Sport Policy Pre-1970, Sport Policy Post-1970, and Sport Policy and Advocacy.

### Policy background

The historical development of Australian sport policy ought to be a robust area of scholarly enquiry, with perspectives of policy development in sport having an essential role in comprehending and informing the evolution of sport policy (22, 37). Despite the increasing importance of government involvement in sport over time (3, 55), however, there has been very little written on sport policy in Australia historically (3, 7), which impacts the sound understanding of sport policy. Indeed, it is suggested that, before 1977, Australian sport history had no formal place in academic institutions, despite the position sport occupied in the national psyche (21, 56, 57). Although “history” refers to the past, the context for Australian sport is essential because “history is actually a bridge connecting the past with the present and pointing the road to the future” (58). Generally, from an historical perspective on sport policy, the federal government in Australia was indecisive in its approach and did not fund sport consistently nor consider it a high priority (3, 59). Equally, academic scrutiny of government policy and Australian sporting activity focused principally on the period from 1970 onwards (3, 7), ignoring policy development and the gradual legitimisation of sport that was surreptitiously underway.

In a nation where sport has, over time, developed powerful connections with the public (3, 60), there is significant literature that addresses the notion that sport was established as a legitimate policy area in Australia in the 1970s (3, 7–14). The prioritisation of sport policy was evidenced by the appointment of a government minister responsible for sport, the establishment of an Australian Sports Council, and significant reviews in sport, including the Bloomfield (59) and Coles (61) reports, and also the recommendation to create the Australian Institute of Sport (AIS), a high-performance centre of sporting excellence (5, 62), and the Australian Sports Commission (ASC), the government agency responsible for developing, supporting and investing in sport at all levels (63, 64). The formation of the AIS reflected a specific government policy to ensure Australia was able to perform successfully on the international sporting stage, a move that resulted in part from Australia's poor showing at the Montreal Olympics in 1976 (3, 5, 21, 65), which was conceptualised three years hitherto (59). Such developments demonstrated the importance of sport and ultimate institutionalisation as a government policy consideration.

The notion of sport marrying with political agendas, particularly state promotion on the international stage, goes some way toward understanding the evolution of sport as an institution. Globally, sport is an institution (66–70), and this is especially true in Australia (10, 20, 71–73), where sport is posited to be fundamental to the nation's society, culture and economy (14, 74–76). Emphasising the social perspective, there is a robust link between participation in sport and the associated societal benefits (77, 78). For example, there have been historical issues of male hegemony creating tension from an education policy perspective and achieving gender balance in sport (79, 80).

Australian schools have long provided the bedrock for many traditional sports like Australian rules football, cricket, netball, rugby league, and rugby union (81). With that structure, there have been issues linked to class and gender inequality, in turn perpetuating the male hierarchy (82, 83). In sharp contrast, there is increasing evidence of the success of female athletes on the international stage where, for example, Australian women repeatedly outperform men at the Olympic Games (81), with the most 2024 Paris Olympics producing the most recent example, with women winning 13 of the nation's 18 gold medals (84). There are other issues that give rise to important policy considerations including societal issues relating to Indigenous peoples which necessitates consideration of endemic problems of racism (81). Policy processes have historically been developed from non-Indigenous perspectives (85) and sport has been used a tool for social control (86).

Noting the non-exhaustive points made about gender, male hierarchy, and the situation impacting Indigenous populations, sport provides a unique platform for social impact and the potential to promote, for example social inclusion and gender equality (87). From a philosophical perspective, sport offers both extrinsic and intrinsic value (88) and benefits such as health, knowledge, skill, and fun (89, 90) and a sense of belonging (91). Within that context, there are policy considerations based on the importance of social inclusion (92) combined with significant physiological benefits for individuals and communities based on participation in sport (93). From a general inclusivity perspective, as evidenced by recent ASC policies such as *Sport 2030*, sport policy in Australia is becoming more inclusive with a focus on the importance of participation, with the government wanting Australians to be “more active, more often” (94).

Significantly, in Australia, sport is viewed as a critical part of the nation's identity (3, 20, 63, 95, 96). Supporting the institutional nature of sport, the ASC resolutely declared that “Sport is one of Australia's great strengths. It is a ubiquitous feature of Australian communities, and its strength and benefits should not be taken for granted or underestimated” (63). Within the Australian sport ecosystem, there are various development and delivery systems (64, 97, 98) facilitated by government, NSOs, and community sport clubs, the latter encompassing sport at the foundational, grassroots level (99). All of the stakeholders in the ecosystem play an essential role in the nation's connection with sport, improving the quality of life and, for example, increasing social capital (76, 100). This situation is, however, only relatively recent from a policy perspective, as it was not always this way.

Before 1970, academic research and commentary in sport had historical and cultural emphasis. There was a dearth of analysis of sport's connection with the government because the federal government adopted an arms-length approach to the creation of sport policy (3, 8–14). Where ostensible government involvement was concerned, literature was scarce concerning policymaking in sport in Australia pre-1970. Stewart et al. (7) make mention of policy developments relating to sport and fitness and, for example, the link to World War II but also point to the generally intermittent approach by the federal government, the relevance being that there was a discernible absence of direct government involvement in developing sport policy. Cashman's (3) *Paradise of Sport*, considered a most influential discourse on Australian sport history (101), discusses aspects of government involvement in sport but notes that some key initiatives were based on foreign policy objectives, for example, deciding where Australian athletes and teams might visit, rather than a robust approach to more general policy. There were further suggestions that sport was a form of foreign policy (102, 103), as opposed to something needing its own policy mandate. Indeed, from an overall perspective, the organisation of sporting activity in Australia in the pre-1970s period was predominantly concerned with non-governmental organisations. Save for small and irregular pecuniary contributions, often as part of foreign policy objectives (102), the federal government largely devolved itself from direct involvement concerning fitness, recreation, and community sport (3, 7), citing responsibility for the activities as reserved for volunteers and the burgeoning sport organisations.

It would be erroneous, however, to infer that the absence of detailed analyses of government contributions to sport policy development meant a relationship was non-existent. Indeed, the pre-1970s era provides an example of government influence on the evolution of sport policy in Australia. In 1911, in the lead-up to World War I, organised physical activity was implemented in schools (5, 104). Further, in 1939, in what was a “significant shift” (7), the federal government's more interventionist role (5) was evidenced by the formation of the Commonwealth Council for National Fitness (CCNF) and the subsequent *National Fitness Act 1941* (5, 13, 105). The period also provided early examples of lobbying and advocacy concerning public health policy (106, 107) and was representative of government policy initiatives with far-reaching consequences, albeit fashioned initially in preparation for war (4, 6, 108, 109).

### Sport policy Pre-1970

Set against predominantly English notions of culture emanating in the nineteenth-century (3, 110, 111), Australian sport was based in part on amateur ideals (112, 113) and British colonial influence (3). Sport offered a way for Britain and Australia to maintain a connection, especially through the creation of equine racecourses and cricket and rugby fields, and ultimately perpetuate social class divisions (81). Such a setting was accompanied by a distinct lack—primarily due to there being no perceived need (7)—of direct or profuse government intervention in sport, especially from a policy perspective (3, 7). Although there was some government investment in sport, such as the provision of open spaces for recreation (114), swimming pools (3), periodic support of international sporting teams (7, 110), and tax concessions for sporting organisations due to their not-for-profit classification (3), it was not until the early twentieth century that the first evidence of tangible and robust policy directives developed.

Although there was a degree of benign indifference behind the evolution of Australian sport policy (7, 17, 22), the threat of the Second World War triggered action by the federal government. Australia's apparent lack of readiness for war provided the urgency for government intervention (3, 108), with the recognition that a policy was needed to develop the nation's fitness levels through recreation and sport (4, 5, 108, 109). The National Health and Medical Research Council (NHMRC) encouraged the government to tackle generally poor fitness levels nationwide (105). The NHMRC Chair, Howard Cumpston, was active in campaigning for change and, as early as 1920, promoted the importance of health and physical fitness (115, 116), a position supported by the federal government (117). The efforts of Cumpston provided an early example of the power of lobbying and advocacy concerning public health policy (106, 107).

At the first NHMRC meeting in early 1937, the necessity to adopt a nationwide fitness program ensued, a suggestion that had the support of the federal Minister for Health (117). Alongside the endeavours of the NHMRC, there was a significant level of effectiveness with political leadership and lobbying, which provided the foundation for the establishment of the CCNF and the ensuing *National Fitness Act 1941* (13, 105). Although funding from the federal government was limited, the Act served as a constructive step toward a program for national fitness (3, 4), and helped provide outdoor activities for schoolchildren (13, 109). It was a considerable initiative for a nation involved in a war that occasioned the creation of public policy requiring a coordinated effort to raise physical activity and fitness levels among the general population (3, 108, 118).

Initially, the federal government founded a National Coordinating Council for Physical Fitness (NCCPF) (119), latterly the CCNF, which was tasked with the creation of fitness programs (1). In concert, the State governments established comparable councils that would function alongside the policy framework developed by the NCCPF (120). With the assistance of federal government funding, these State councils would, in turn, coordinate policy implementation with local government to facilitate community programs. It was the first successful effort—as history would reveal (17)—to engage three levels of government in a coordinated, synchronised manner, notwithstanding that the prime objective was to improve the fitness of military personnel (17, 108).

The CCNF broadened its reach and influence to address health and fitness initiatives nationwide and across various demographics (4). The development of the CCNF was buoyed by examples of further and persistent lobbying and the acknowledgement of the service provided, particularly where it complemented State government initiatives (17). Services included establishing sport, recreation, and fitness amenities, including sports centres and green space for outdoor activities (4, 109). The range of activities and stakeholders involved developed the notion that the private sector and the local community should be engaged with and influence government policy on developing fitness, recreational, and sport initiatives (120).

During this developmental period, the CCNF did not provide direct support, either by way of funding or policy input (17). Nonetheless, many fitness and health programs supported by the CCNF in the years that followed directly impacted sport policy, particularly from the perspective of lobbying (106). Although the focus of the CCNF had initially been on fitness and health, organised sport was gradually added to the policy objectives (4). Indeed, with fitness and health as the ultimate goals, organised sport served as a means to that end (121, 122). Thus, fitness, health, recreation, and sport fused to form part of the same policy continuum, a process initially reliant upon local councils, as opposed to Federal government intervention (3).

The priorities for the CCNF focused on aspects other than the war, including adopting a role separate from the fitness of military personnel. In 1941, the then Minister for Health suggested that we “must not forget the ultimate goal of fitness to enjoy life” (120). Although funding was limited in the initial stages, expenditure was directed with intent; there was an emphasis on funding physical education in schools and establishing tertiary qualifications nationally (120). Indeed, there was a clear requirement for physical education programs to be tailored to government and CCNF objectives (1, 3). In addition, the CCNF broadened its influence to encourage the creation of physical education faculties at tertiary institutions, physical education programs in schools, youth fitness strategies, and related research (3, 105, 123), all precursors to the overall goal of a healthier nation as part of improving sport policy (3, 17, 109).

Concerning community sport policy development, achieved mainly through providing recreational facilities in the post-war period (65, 124), this formed part of CCNF policy agendas and was loosely coordinated with Australia's NSOs (3). The policy considerations developed exponentially as the CCNF became increasingly influential, particularly post-war, with significant advocacy for establishing playgrounds, for example (104, 105). The fitness movement developed beyond the war-related impetus, and CCNF involvement increased interest in sport policy from the government (4). Equally, NSOs sought more significant input from the CCNF, and elite sport emerged as an item for discussion and decision, especially in the years leading up to the 1956 Olympic Games in Melbourne (17).

Sentiment toward high-performance sport was not, however, entirely positive. For instance, some State governments expressed apprehension toward supporting minor sports that were not participating in the 1956 Olympic Games (4). The concern related to resources being used to improve high-performance sport, which countered the spirit of sportsmanship with an emphasis on winning (125). Indeed, the dominant agenda in political circles was that sport should not be the responsibility of the government (126). Although the CCNF was involved in various activities targeting fitness and in 1967, for example, then Prime Minister Harold Holt enacted the “Fitness Australia” campaign (105), there was a disparity between approaches to general health and sporting success. Hence, State government investment in sport during the 1950s and 1960s was limited and Federal government support for international events was “ad hoc” (5). The view was that sport was antithetical to traditional policy involvement by the government (127, 128); the opinion was that non-utilitarian, private leisure pursuits were not an area for government (5, 126). Consequently, volunteers in the local community, with the help of local government, took on the duty of fundraising (1).

Overall, government funding for sport was erratic leading up to the 1970s, and significant funding from State governments was limited (128, 129). Although the 1950s and 1960s were considered a period of growth for Australian high-performance sport (126), before the 1970s, successive federal governments were relatively indifferent—having a benign attitude (7, 17, 22)—toward funding sport, viewing international competition as the only area worthy of substantial investment (13, 16, 17). That said, putting aside the focus on the readiness for war, the advent of the CCNF paved the way for the federal government's burgeoning partnership with other levels of government and influence on community sport policy through lobbying (106). On a rudimentary level, the CCNF outlined the policy requirements for the government to support the health and fitness of the nation and provide opportunities for Australians to improve their overall well-being. The CCNF role developed slowly to support sport at grassroots level, including the need for facilities, coaching, and organisational sustainability (4). In addition, the *National Fitness Act 1941* encouraged engagement across all levels of government and ensured fitness featured in the more macro public health policy considerations (13, 105, 130).

### Sport policy post-1970

From the perspective of the development and, crucially, evaluation of Australian sport policy, the 1970s was a period of significant change. There were fundamental political positions to address concerning sport and recreation strategies, namely, to assess the level of involvement of the federal government in policy and the Australian sport system (3, 7, 129). The decade involved what was an increasing level of policy discourse and the influence and evolution of policy practices (131), which included a more formal approach to sport policy, with a strong focus on elite sport and a lesser focus on sport participation policies (37, 132). Politically, and no less from a sport policy perspective, the 1970s was tumultuous. The pioneering work of the new government from 1972 to 1975 was followed by a period of stagnancy by the incoming government in November 1975 (7, 133). From an overall standpoint, however, the political and the sport systems were arriving at a point of unison from the perspective of the nation's health (33, 64, 134). As the Prime Minister described, “There is no greater social problem facing Australia than the good use of expanding leisure … so we must prepare now; prepare the generation of the 80s—the children and youth of the 70's to be able to enjoy and enrich their growing hours of leisure” (135). There was the pursuit of a new and more interventionist role in sport policy, in both high-performance and community sport (128).

As part of a relatively new agenda—where the federal government significantly raised funding for sport to levels far higher than previous governments (136)—a Tourism and Recreation office was created. The new department initiated a program intended to increase the number of sports facilities, established a program to support NSOs, and launched a national fitness awareness campaign (108). The Australian Sports Council was established (137), and several separate and ground-breaking reports and inquiries into sport and recreation were commissioned, including the Coles Report (61) advocating for the establishment of the AIS (3, 8, 9, 12, 14). It was a period of increasing political engagement with policy architects demonstrating a burgeoning interest in sport and, in turn, greater involvement with the sports organisations that formed the Australian sport ecosystem (3). A 1973 Department of Sport and Recreation report entitled *The Role, Scope and Development of Recreation in Australia* outlined government involvement in sport in four areas: (i) broad policies underlying the development of recreation in Australia; (ii) recreation; (iv) sport and fitness; and (iv) training and research (59). The report essentially was adopted as a “blueprint” for guiding government policy on sport from the elite level to community sport (138).

Additional rationale for government involvement, at both federal and state levels, included using sport to improve employment opportunities, encourage tourism, enhance image and popularity, and increase revenue (3). As part of policy consideration during this period, the commercialisation—or “hyper-commercialisation”, as suggested by some (7)—of sport was based on changes to leisure and recreation that occurred because of broader developments in capitalist countries (7). This development tied in with, in some sectors of sport, the end of amateurism, where Australia “jettisoned its English model of sport for a more commercial one” (3), resulting in significant influence on sport by the forces of commercialism (134, 139). For example, the advent of live television broadcasts created a more overt commercial focus on sport where professional modes of sport impacted historically amateur structures (81). With such commercial developments, however, came an increasing level of influence on high-performance sport that gradually began to erode foci on policy development related to health and sport in general (18, 140).

Commercial developments aside, a key policy objective of the Australian Sports Council was to provide the federal government with “expert advice on the development of sport and physical recreation in Australia, to encourage mass participation and develop excellence” (141). Such advice, based in part on examining the international environment and studying sporting structures in comparably-resourced countries, was considered the most realistic avenue for the government to obtain sound guidance on developing sport policy (142). Accordingly, the Australian Sports Council became the first organisation of note to advise and lobby the federal government on sport policy. Similar structures in predominantly Western nations across the globe were assessed as part of the initiative, save that East German and Chinese sport academies were also examined (3, 7). It was apparent that several nations possessed more advanced sport policy frameworks than Australia and were “actively engaged in fostering the participation of the public in recreation activities” (141). Mindful of these developments, the minister responsible for sport emphasised the commitment to general welfare, noting that the government acknowledged responsibility for boosting the nation's opportunities for recreation and physical well-being, but adding that “much more needs to be done” (143). This comment concerning the nation's welfare needs to be considered in terms of advocacy for the value of sport and incorporate the impact of cultural influences on Australian sport. Indeed, in what amounts to counter-productive examples of advocacy, the influence of powerful alcohol, tobacco, and gambling lobbyists in conjunction with the commercialisation of sport has significantly impacted the Australian sporting landscape (40).

### Sport policy & advocacy

In the first instance, some historical context relating to how advocates for alcohol, tobacco, and gambling have influenced Australian sport is provided. Alcohol had a predominant link to sporting activities in the nineteenth century and created a culture common in Australia today. Originating in popular sports like horse racing and boxing, alcohol was a significant focus for nineteenth-century sporting events (144–146), and such influence only strengthened in the twentieth century (7, 147). With the increasingly commercialised nature of sport, the alcohol industry was able to sponsor sporting events, a development that became standard practice in Australia, perhaps even more so than elsewhere internationally (148, 149). The smoking lobby has a long, historical connection with sport, mainly due to the once popular view that it was “acceptable and fashionable for sporting males to smoke” (3) There were early examples of advocacy endorsing smoking; for example, the Rothmans National Sports Foundation, founded in 1964, engaged professional athletes as part of its campaign to lobby the government (3). Gambling is synonymous with Australia's popular culture due to a relatively liberal approach to the phenomenon, leading to gambling becoming part of general lifestyles (150). The industry is very profitable, heavily involved in sport sponsorship (151), and makes significant contributions to government revenue (152, 153). Overall, there are suggestions that in Australia, there is a lack of transparency relating to lobbying connected with tobacco, alcohol and gambling firms, and with that comes the potential for commercial interests to have undue influence on the formulation of health policy (40).

Conversely, from the perspective of advocacy targeting the promotion of welfare through sport by positively influencing health policy, the components linking lobbying and sport policy in Australia were, historically, largely informal and variable in terms of influence. Even with a greater government focus on sport policy in the 1970s and early 1980s, advocacy groups lacked influence and sport “demanded little in return for political exploitation” (154). Although there were historical examples of lobbying concerning sport policy (e.g., Cumpston and the NHMRC, and the CCNF), there was no formal method or organised group to advocate for Australians to, for example, gain increased access to sporting events. The lack of formal advocacy was at a time, however, when there were moves to create a national sporting event alongside the consideration of a formal process for campaigning for sport. The dearth of high-level competition was a consideration for the Australian Sports Council, which discussed options for athletes to participate in a national event involving various sports, accompanied by the development of international standard stadia. Raised by Bloomfield in 1973 and dubbed the “Australia Games”, the Australian Sports Council supported the concept, but the endorsement was subject to the concurrent creation of a sport confederation to influence the development of Australian sport policy (155).

The recommendation for creating a sport confederation was significant because it highlighted an emerging framework for sports policy in Australia (64, 156) and that establishing such an organisation would encompass the affiliation of the nation's NSOs and campaign for the interests of all levels of sport. Incorporated as the Confederation of Australian Sport (CAS), the organisation was positioned as the dominant national advocacy group, with the objective of lobbying the federal government for funding across sports (3, 64). This development was fundamental in the nation's sport policy landscape and further legitimised the increasingly institutional nature of Australian sport. The formation of CAS provided an independent voice for the Australian sporting industry, “designed to express opinions on Federal Government deliberations and decisions on sport” (3). CAS sought to work closely with NSOs and was committed to supporting organisations responsible for developing community sport. The historical background to the organisation's creation followed the government's judgment that there was a significant appeal in having a group able to advocate on behalf of essentially national health-related policy interests linked to sport (4). The government gave strong backing to a sports confederation that supported national objectives, including appropriate levels of investment in sport. The benefits of sport and the value of a lobby group acting on behalf of national sporting interests were promoted by the then minister responsible for sport, who stated: “… I would like to present the case for a pressure group, even if this group, for a change, is the vast majority, the silent majority of our country …” (143).

CAS was not fully established until 1976, with one key impetus for creating the organisation being Australia's dismal performance at the 1976 Olympics in Montreal (3, 5, 21, 65). Just prior to the official formation of CAS, there was a change of government in 1975 and a significant policy shift resulting in reduced funding for sport (4, 15). This situation produced further momentum for CAS, partly due to the connection between government investment and success at international events (157).). Within that setting, it was clear that NSOs needed to address funding shortfalls, which provided legitimacy to CAS regarding its role in advocating for NSOs. The change in government increased high-level discourse between NSOs, CAS and the federal government, further justifying the formation of CAS. Founded initially by forty-two sport organisations, by 1985, CAS had 123 affiliates representing circa six million Australians who were members of an estimated ten thousand sporting clubs, thus giving CAS “political clout” (15).

CAS relocated to Canberra to mix more efficiently in political circles and to better influence government decisions. The main challenge for CAS was how to impact what the government might ultimately choose to do, an issue associated with public policy (158) and specific characteristics of sport that results in a different regulatory perspective (159), as evidenced by the historical background to government involvement in sport policy. CAS focussed on collaboration, advocacy, and service to NSOs and community sport concerning government policy. Although funding for sport was an essential agenda item, CAS was more than a campaigner for pecuniary assistance; it also sought to promote the merits of more general health-related goals (154). Lobbying was CAS' “*raison d’etre*” (15), and the organisation's objective was to understand how and where decisions were made and how best to influence them (15).

Although the relationship between CAS and the government was complicated, sport did become a more serious government consideration (5, 15, 21). Indeed, for some years post-1976, CAS developed a significant presence in shaping government sport policy by challenging government strategies and lobbying for increased funding for sport. The 1980s, in particular, represented an era that witnessed the “emerging sophistication of the sports movement itself, through the numerous national sporting bodies and their constituents” (16) and when CAS had a significant role in promoting the need to improve sport policy. The organisation's overt activity in championing the requirements for sport on a national and international level was a catalyst for what, in effect, was a more expeditious advancement of the sport policy process. The 1980 CAS white paper—*A Master Plan for Australian Sport*—sought to ensure that government policies on sport served both elite and community objectives (7) and echoed aspects of the Bloomfield Report calling for a national sport policy that encompassed all levels of sport, including school sport (65, 160). Overall, CAS was part of—and contributed to—an era where the government had a responsibility to support the various stakeholders that made up the Australian ecosystem (15). Bloomfield's notion of the Australia Games was eventually fulfilled by CAS with the inaugural (and only) event occurring in early 1985 (155).

Despite a robust presence historically, however, CAS was forced to review its performance, redefine its priorities, and set new standards on more than one occasion (15) as it adjusted to strategic shifts. CAS's usefulness, however, waned over time. Notwithstanding being the national voice for sport, the actuality is that CAS has lacked relevance more recently (161). Moreover, advocacy groups, in general, have proved ineffective in terms of sustainability in community sport. Seemingly unintentionally, they serve a limited purpose for a finite period or lack the resources and independence to provide adequate guidance and support at grassroots level. Indeed, in what amounts to a troubled history, there is a pattern of early success, limited impact, and occasional redundancy.

By way of other examples, ACTSport, the peak body representing sporting associations in the Canberra region, ceased trading in 2015. According to the announcement on the organisation's now-defunct website, the sport industry has matured, sport has changed, communications have changed, and how sporting organisations do business has altered markedly—thus, the perceived need for a sport advocate declined. ACTSport had only a minor role in sport in the ACT and only focused on the Hall of Fame and, at the time, a start-up exposition that is no longer in operation. As a further example, SportNSW, an independent member-based peak body representing NSW as an advocate so that more people can benefit from sport (162) is somewhat detached from the community. Although it purports to represent the interests of community sport, the actual influence is limited. As a final example, Sport Central Coast purports to be the voice for sport for the NSW Central Coast and provides a database of sporting clubs in the region. Based on preliminary observations, however, all indications are that the organisation has perhaps a limited impact and, save an annual sports awards ceremony, owing ultimately to resource issues, offers less than it intends. There are other examples of groups advocating for sport, such as Disability Sport Australia, but these are largely specialist/targeted and do not offer a universal approach to sport policy as CAS once did.

From a professional sporting perspective, the Coalition of Major Professional and Participation Sports (COMPPS) is a self-funded lobby group representing seven professional Australian NSOs as its core membership. Formed in 2010, COMPPS seeks to promote the collective interests of its members and references sport as a significant factor in the social and economic well-being of the nation. COMMPS has been active with a community focus, in part as a reaction to catastrophic situations such as the 2020 bushfires (163, 164) and, more recently, the impact of COVID-19 on community sport (165, 166). A cursory search, however, reveals that COMPPS is primarily concerned with issues such as corruption, gambling, drug use, match-fixing, alcohol licensing, gender diversity, and religious discrimination [see (167–173)]. Further, while the organisation suggests that it invests heavily in sport for all Australians, of the over sixty government submissions from COMPPS since its inception, save for two recommendations resulting from the COVID-19 crisis, only two—the *Active After School Program* submission, 2011, and the *National Infrastructure Audit* submission, 2019—relate directly to participation sport. Also, and not insignificantly, sports that are not members of COMPPS sports received far less government funding, partly due to their lack of lobbying power (174).

In contrast, the lobbying activities of COMPPS point to a powerful presence in key commercial areas, namely alcohol and gambling sponsorship. From a macro perspective, the alcohol lobby in Australia occupies a formidable position. In 2023, the alcohol lobby ploughed $1.3 million into political parties in 2023, which is contrary to the wellbeing of the community (175) and also recently challenged the World Health Organisation's attempts to reduce alcohol consumption rates by 2030 (176). Noting the connection between alcohol sponsorship and sport, COMPPS suggested that reducing the opportunities for Australians to engage with alcohol would damage the pecuniary aspects of Australian professional sport irreparably (167). Further, with the support of recent academic research, it is evident that alcohol industry lobbyists misrepresent evidence to influence government policy in Australia (177, 178) and internationally (179). Regarding gambling, Australian sporting codes recently rejected the need for additional regulation of online gambling (180). Through their representative body, COMPPS, it was suggested that a balance between protecting children and catering for legitimate betting could be arrived at, a position derided by various politicians and the Alliance for Gambling Reform (181).

**Figure 1:**
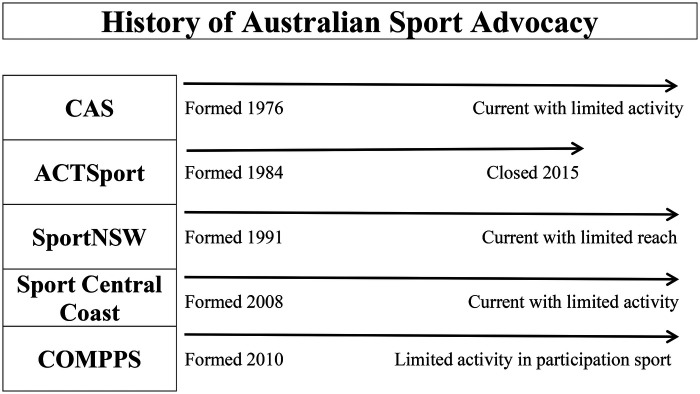
History of Australian community sport advocacy.

In that context, it is evident that COMPPS occupies an awkward position in the Australian sport ecosystem and, indeed, offers an example of marginalisation where the commercial interests of a select group of professional NSOs take precedence over sport in general, including the idea of increasing participation in sport across the nation. Indeed, there have been anecdotal suggestions that the second “P” in the COMPPS acronym—standing for “participation”—was a token gesture on the part of the organisation and is of little significance to their ultimate commercial objectives. Based on the conflicted nature of the COMPPS approach to sport policy, where it does not act in the interests of sport across that nation, the void in terms of advocacy for all sports, and all levels of sport, is evident. Indeed, overall and as outlined briefly in Figure 1, there is a common theme evident where CAS, ACT Sport, Sport NSW, and Sport Central Coast are concerned. These four entities demonstrate strategic issues and in the case of COMPPS, there is an example of marginalisation where professional sport takes precedence over community sport.

## Discussion

By way of a brief synopsis of the findings, this paper provides a narrative of the sport policy development process in Australia, along with the development of advocacy in sport. From an historical perspective and based classification in four key themes, the research outlines policy background, then illuminates government policy objectives prior to the 1970s, followed by a sharp change from the 1970s onwards, along with the advent of lobbying in sport. On this latter point, however, there is a degree of organisational complexity relating to sport policy, including establishing the potential for constructive—and sustainable—relations between government and advocacy groups (15, 64, 182). Further, the sport policy domain lacks precision regarding the influence and structure of lobby groups, especially in Australia. What might have once been a pathway for a universal policy system supporting all forms of sport, underpinned by the advocacy of CAS, has become a fractured policy system where numerous stakeholders lobby for resources for their area of interest (134), as evidenced by COMPPS. The tenuous situation is magnified by the holistic focus of some stakeholders whose primary focus is on sport facilitating wider social benefits—sport as an end in itself (183–185)—versus organisations more concerned with commitment to sport for other reasons—as a means to an end (121, 122).

Such challenges are apparent in Australia, where the federal government has, since the 1980s, been regularly at odds with policy priorities for sport, particularly when evaluating elite sport policy alongside participation policy (64, 129, 186, 187). Although policies focussed on investment in elite sport and associated events have been well publicised in Australia (188), significant conflicts arise about policy priorities relating to negative trends linked to participation sport (189–191). The ASC was, in part, set up to encourage greater participation in sport and recreation for all Australians but has faced an ongoing dilemma regarding its approach to participation (33). In particular, there have been problems with the measurement of mass participation data (33) in terms of the impact of policy objectives at the elite level and what has amounted to a limited “trickle-down” effect (5, 55, 129, 192).

Although the ASC has taken steps to analyse better and act upon participation data (14) and has enacted policies (e.g., the *Sport 2030* plan and the *Play Well* strategy) that adopt a more comprehensive approach to sport and the mitigation of health issues through physical activity (33, 193), aspects of the government's approach have failed. Indeed, contrary to the edicts of the *Sport 2030* plan, the federal government soon abandoned direct support and funding for community sport (194). Although limited in lobbying power, CAS put forward its conceptual support for the *Sport 2030* plan as an example of cross-sector collaboration (195, 196). The government's rejection led to a response from CAS that made its concerns clear and that the step reversed one of the key pillars of *Sport 2030* (197). Such vacillations impacting community sport programs are demonstrative of a general inability of the government to arrive at a uniform and consistent policy for sport in general (3). Without independent and robust advocacy, policy creation will likely remain a top-down process and inconsiderate of some key stakeholders.

### Future direction

A successful sport policy system requires stakeholder cooperation; a robust nexus between all levels of government, NSOs, sport administrators, academia, and community sport organisations (64). Further, there is a need for a policy process based on clear governance structures that facilitate a sincere and practical national policy focus (33). In order to exert influence on a complex sport policy environment, an understating of the beliefs of stakeholders where underlying conflicts in a coalition focussed on participation sport are increasingly common, hence the concept of a coalescent approach to advocate on behalf of sport is significant (129, 198, 199). Advocacy coalitions are characterised by stakeholders possessing similar or aligned beliefs and collaborating to impact policy to their advantage (200). Advocacy coalitions of this nature, viz., an alliance of stakeholders with analogous beliefs and operating with policy consensus, increase the probability that such groups will influence policy change (201, 202). From the perspective of addressing beliefs through a coalition, stakeholders will invariably connect with others with comparable objectives, such as COMPPS, thus facilitating a united and cohesive position to address their strategic objectives (182, 203).

To test the merits of the above concept, as a guide for further research, there is an opportunity to combine questionnaires, interviews, and other empirical research methods, with a predominantly systematic methodology. Adopting a mixed methods approach to policy research, based on a combination of quantitative and qualitative longitudinal data, is “potentially very powerful in providing links between causation, processes and outcomes” (204). Obtaining first-hand data and conducting empirical analysis can increase the research conclusions’ depth and persuasiveness. Such an approach has been undertaken, albeit on a limited scale, in regional Australia, and involved a survey of community sport clubs, in-depth interviews with senior community sport club officials, followed by an intervention in a regional town [see (182, 205, 206)]. The latter aspect to the research provided useful data relating to the potential for bottom-up policy influence and provides an outline for further, more extensive research, especially in relation to assessing the potential of sport advocacy. In so doing, the objective would ultimately point to a sport policy process that was more inclusive and considered the views of a greater, more representative range of stakeholders. Through greater collaboration, the likelihood of policy development and improvement has potential where government and NSO policies are modified due to stakeholders amending their perception of existing policies (37, 207).

Aligning policy beliefs through a collaborative approach to an advocacy group's central priorities and identifying wellbeing as the most significant concern is crucial, albeit challenging given the commercialised nature of professional sport. In the sport policy domain, there is also the battle relating to the varying levels of commitment from the government to elite sport vs. community sport (191). The challenges of finding a balance for the varying levels of sport and the application of policy frameworks is well documented (37), but there are also suggestions for approaches that incorporate consideration of existing policy frameworks that incorporate a greater range of stakeholders (43, 208) Indeed, an inclusive, holistic, unique, and all-encompassing sport policy framework has merit (38, 39), provided there is a willingness from key stakeholders to collaborate on the principle of universal well-being. From a slightly ironic perspective, CAS made similar suggestions before the turn of the twentieth century when it called the consideration of a sport policy framework that might apply going forward and the “principles, priorities, and policies around which it should be constructed” (156). CAS added that a workable framework requires articulating and creating opportunities for “collaboration within the Australian sporting community in its evolution and development” (156).

With the above context, an international example of advocacy in sport provides detail of the complexities of institutional structure and legitimacy. The Sport and Recreation Alliance (SRA), the organisation representing the National Governing Bodies (NGB) of sport in the United Kingdom, represents three-hundred-and-twenty sporting organisations. The SRA advocates for all its member organisations, providing services that assist with structured organisational management, including grassroots sport. The SRA faced some strategic issues similar to those that plagued CAS, but it provides an example of an advocacy group successfully re-establishing itself after 2010. There was a range of issues including (i) poor leadership and apathetic staff where decisions and strategies were poor, and there was a lack of empathy with government objectives; (ii) an absence of connection with membership in general and with the more modern NGBs; (iii) lack of respect; (iv) lack of visibility; and (v) a poor relationship with Sport England, the UK equivalent to the ASC, an organisation that raised issues of non-performance, and cynically controlled funding to put pressure on the SRA.

To address these issues, the SRA (i) introduced new and better-qualified leadership, including the CEO and Head of Governance and Policy; (ii) created a new Board comprised of individuals willing to consider change and support the Executive; (iii) ensured the Executive had the trust of the Board and encounter less bureaucracy implementing programs; (iv) reduced staff turnover and when necessary, hired highly qualified, and dynamic employees; and (v) enacted a complete restructure in 2015 to ensure organisational sustainability for the foreseeable future. From a governance perspective and the objective of achieving “best practice”, the SRA emphasises a collaborative approach to the administration of all levels of sport but with a strong focus on grassroots sport (209).

## Conclusion

Sport policy in Australia has a long but erratic history. Despite an increasing level of government interest over the course of the twentieth century, effective and sustainable policies that achieve universal outcomes remain out of reach. The absence of an organisation lobbying for an holistic, national sport policy leaves the Australian public subject to inconsistencies in policy application and undue influence from organisations prioritising commercial success over health policy. Future policy directives must prioritise the nation's welfare over the pecuniary interests of dominant professional sports. Based on the recommendations for further research, a more inclusive and harmonised approach to policy formation through an advocacy structure provides direction for informing and guiding the overall policy process. An independent lobby group with clear strategic objectives and a mandate encouraging collaboration that effectively combines government health objectives with a robust policy for sport is a worthwhile consideration. There are many challenges to facilitate such an objective and will be met with “pushback”—benign indifference—from certain quarters. Indeed, lobbying for the well-being of all will be perceived as a direct challenge to the overt commercial interests of certain professional sports. As history shows, such an approach might be fashioned in preparation for a different kind of war.

## Data Availability

The original contributions presented in the study are included in the article, further inquiries can be directed to the corresponding author.
